# The genetic background and vitamin D supplementation can affect irisin levels in Prader–Willi syndrome

**DOI:** 10.1007/s40618-021-01533-4

**Published:** 2021-03-03

**Authors:** M. F. Faienza, G. Brunetti, G. Grugni, D. Fintini, A. Convertino, P. Pignataro, A. Crinò, S. Colucci, M. Grano

**Affiliations:** 1 Department of Biomedical Sciences and Human Oncology, Section of Pediatrics, University of Bari ‘A. Moro’, Bari, Italy; 2 Department of Biosciences, Biotechnologies and Biopharmaceutics, University of Bari ‘A. Moro’, Bari, Italy; 3 Division of Auxology, Istituto Auxologico Italiano, Research Institute, Verbania, Italy; 4 Endocrinology Unit, Pediatric University Department, Bambino Gesù Children’s Hospital, Rome, Italy; 5 Department of Emergency and Organ Transplantation, University of Bari ‘A. Moro’, Piazza Giulio Cesare, 11, 70124 Bari, Italy; 6 Department of Basic Medical Sciences, Neuroscience and Sense Organs, Section of Human Anatomy and Histology, University of Bari ‘A. Moro’, Bari, Italy; 7 Reference Center for Prader–Willi Syndrome, Bambino Gesù Hospital, Research Institute, Via Torre di Palidoro, Palidoro, Rome, Italy

**Keywords:** Prader–Willi syndrome, Irisin, Vitamin D supplementation

## Abstract

**Background:**

Prader–Willi syndrome (PWS) is associated to distinctive clinical symptoms, including obesity, cognitive and behavioral disorders, and bone impairment. Irisin is a myokine that acts on several target organs including brain adipose tissue and bone. The present study was finalized to explore circulating levels of irisin in children and adult PWS patients.

**Methods:**

Seventy-eight subjects with PWS, 26 children (15 females, mean age 9.48 ± 3.6 years) and 52 adults (30 females, mean age 30.6 ± 10.7) were enrolled. Irisin serum levels were measured in patients and controls. Its levels were related with anthropometric and metabolic parameters, cognitive performance and bone mineral density either in pediatric or adult PWS. Multiple regression analysis was also performed.

**Results:**

Irisin serum levels in PWS patients did not show different compared with controls. A more in-depth analysis showed that both pediatric and adult PWS with DEL15 displayed significantly reduced irisin levels compared to controls. Otherwise, no differences in irisin concentration were found in UPD15 patients with respect to controls. Our study revealed that in pediatric PWS the 25(OH) vitamin-D levels affected irisin serum concentration. Indeed, patients who were not supplemented with vitamin D showed lower irisin levels than controls and patients performing the supplementation. Multiple regression analysis showed that irisin levels in pediatric and adult PWS were predicted by the genetic background and 25(OH)-vitamin D levels, whereas in a group of 29 adult PWS also by intelligent quotient.

**Conclusion:**

We demonstrated the possible role of genetic background and vitamin-D supplementation on irisin serum levels in PWS patients.

## Introduction

Prader–Willi syndrome (PWS) is a rare genetic disease with distinctive clinical symptoms that critically impair patients’ quality of life. PWS arises because of the lack of expression of genes located on the paternal chromosome 15q11.2–q13. Three main genetic mechanisms have been recognized in determining PWS: interstitial deletion of the proximal long arm of chromosome 15 (del15q11–q13) (DEL15), maternal uniparental disomy of chromosome 15 (UPD15), and imprinting defects [1]. The main features of the PWS phenotype are broader, and include neonatal hypotonia, poor feeding and initial failure to thrive, followed by hyperphagia and early childhood-onset obesity (if uncontrolled), multiple endocrine abnormalities (including growth hormone deficiency (GHD) and hypogonadism], motor development problems, dysmorphic features, cognitive impairment, and behavioral issues [2]). Notably, PWS patients also show bone impairment. In detail, prepubertal PWS children display normal bone mineral density (BMD) (if adjusted for the reduced height) [3–5], but in adolescence and adulthood, they presented decreased total BMD and bone mineral content (BMC) possible, because they did not achieve bone mineral accrual, also due to pubertal delay/hypogonadism [6–9]. Consequently, osteoporosis is predominant in PWS individuals, who also have other orthopedic complications related or worsened by weight gain, including scoliosis, kyphosis, hip dysplasia, flat feet, genu valgum and fractures [8, 10].

Recently, researchers have shown an increased interest on irisin, a myokine primarily secreted by skeletal muscle, involved in bone, adipose tissue and brain homeostasis. In detail, in young mice irisin injection mimicked the effects of exercise by increasing cortical bone mass and strength [11]. In hindlimb unloaded mice, intermittent administration of irisin prevented bone loss [12]. Interestingly, the myokine is also known to determine the browning of white adipose tissue [13] and to work as an adipokine as it is secreted by the same tissue [14]. Additionally, it has also been reported that irisin may have an effect on certain brain functions, and consequently it is involved in cognitive impairment and in neurodegenerative disease [15, 16]. These issues prompted some authors to evaluate irisin levels in adult PWS patients. In detail, Hirsch et al. found increased amounts of salivary irisin in obese PWS with respect to non-obese controls, whereas the plasma levels of irisin did not change significantly between the two groups [17, 18]. Recently, Mai et al. also reported that PWS patients and controls have similar circulating irisin levels [19]. The present study was finalized to explore circulating levels of irisin in children and adult PWS patients in relation to the genetic background, metabolic profile, cognitive impairment and bone status.

## Materials and methods

### Patients

Seventy-eight subjects with PWS, 26 children (15 females, mean age 9.48 ± 3.6 years) and 52 adults (30 females, mean age 30.6 ± 10.7) were included in this study. All patients showed the typical PWS clinical phenotype [20]. Genetic investigation was performed in all PWS patients, and 48 of them had DEL15 (32 adults and 16 children), while UPD15 was found in the remaining individuals (20 adults and 10 children).

All PWS children were on growth hormone (GH) treatment from at least 12 months, at a dosage ranging from 0.025 to 0.035 mg/kg/day. Among PWS adults, 6 out of the 52 subjects presented a severe degree of GHD, according to a GH response to GHRH plus arginine less than 4.2 ng/ml [21], and received GH therapy at a mean dose of 0.23 mg/day. At all ages, the GH dose was adjusted to maintain serum total IGF-I within 2 SD from an age-matched reference value to avoid overdosing. At the time of the study, 10 females and 1 male underwent sex steroid replacement treatment.

As controls, we evaluated a group of 26 children (17 females, mean age 9.4 ± 3.29 years), referred to our hospital for minor surgery or electrocardiographic screening, and 54 normal weight adults (26 females, mean age 36.5 ± 12.5 years) enrolled on a voluntary basis.

PWS and control children performed an average of 2 h per week of school sports, whereas adult PWS and controls about 3 h per week. Four out of 26 PWS children (15%) and 26 out of 52 PWS adults (50%) were on vitamin D supplementation at the moment of the study [cholecalciferol mean dosage: children 500 UI/daily (12.5 mcg/daily); adults 800 UI/daily (20 mcg/daily)].

Exclusion criteria from the study for both patients and controls were the use of mineral and vitamin supplements, except for vitamin D, the presence of chronic diseases with a possible impact on bone metabolism (e.g., hypothyroidism or hyperthyroidism, Cushing’s syndrome, celiac disease, anorexia nervosa, etc.), the use of medications affecting bone turnover, e.g., corticosteroids, and fractures in 6 months preceding the study. Five adult PWS patients had a history of previous post-traumatic fractures at different sites (ankle, ulna, radio, malleolus, fibula, and phalanges). None of the PWS pediatric patients experienced fractures.

No patient had previously undergone bariatric surgery.

Written informed consent was obtained from all the legal guardians, and from the patients when applicable, prior to inclusion. All procedures were approved by local institutional review boards.

### Anthropometric measurements

All patients underwent a general clinical examination, anthropometric measurements (height in cm, weight in kg) and, for the pediatric age, data were plotted on Italian growth charts and computed as percentiles and SDS [22]. BMI was defined as weight in kilograms divided by the square of height in meters. The international standards for sex- and age-specific BMI percentiles were used for subjects aged 2–18 years [22]. BMI standard deviation score (SDS) was derived from the published Center for Disease Control and Prevention (CDC) standards [23]. The BMI cut-off point of > 2 SDS was used to define obesity, and between 1.4 and 2 SDS to define overweight for individuals < 18 years of age. Considering adult age, we considered as obese, overweight and normal-weight those subjects with a BMI > 30, in the range of 25–30 and < 25, respectively (NIH). The pubertal and genital stages were assessed according to the Tanner criteria [24].

### Biochemical measurements

Blood samples were drawn under fasting conditions, centrifuged, and stored at − 80 °C until required. Blood glucose, insulin, total cholesterol (TC), high (HDL) and low (LDL) density lipoprotein cholesterol, triglycerides (TG), were measured after overnight fasting in all subjects, using standard methods. Values of TC, LDL, HDL, and TG were considered in the normal range if within the 5th and the 95th percentile. Calcium, phosphorus and alkaline phosphatase (ALP) concentrations were measured by the nephelometric method. Serum active intact parathyroid hormone (PTH) and 25(OH) vitamin D were measured by immunological tests based on the principle of chemiluminescence using commercial kits (Liaison assay; DiaSorin, Stillwater, Minnesota, USA). Osteocalcin serum concentration was measured by enzyme immunoassay (IBL International GmbH, Hamburg, Germany). Irisin levels were assessed using a commercially available kit (AdipoGen, Liestal, Switzerland). Insulin resistance was assessed calculating the homeostasis model assessment (HOMA) [25].

### Dual-energy X-ray absorptiometry (DXA)

Bone mass of the anterior–posterior lumbar spine (L1–4) and the total body (TB) was measured by DXA using a Hologic QDR Discovery, and the APEX-system software version 13.3 (Hologic Bedford, MA) with fan beam in array mode. The measurements were performed using standard positioning techniques. Quality control scans were performed daily using a simulated L1–4 lumbar spine phantom. The lumbar spine DXA scan were analyzed to generate measures of L1–L4 vertebral areal BMD (LSBMD, g/cm^2^), bone mineral content (LSBMC, g), spine volumetric BMD (LSBMAD, g/cm3) lumbar spine *Z* score (LSBMD *Z* score, SDS) [26–28].

Total body scans were obtained to estimate fat mass (FM%), fat free mass (FFM%) expressed as percentage of total body weight (bone mass with the skull excluded from analysis (total body less head, TBLH). Bone variables included BMD (TBLH BMD, g/cm^2^), BMC (TBLH BMC, g); BMD were normalized for height (TBLH BMD-Ht, g/cm^3^) to avoid any influence of growth on bone mass [28].

### IQ assessment

Global IQ evaluation was assessed in a subgroup of patients based on age. In details, in pediatric PWS it was used Wechsler Intelligence Scale for Children-IV (WISC-IV, *n* = 6) [29] or Leiter International Performance Scale (*n* = 5) [30]. The Wechsler Adult Intelligence Scale-IV (WAIS-IV) was used in 29 PWS adults. This test allowed the calculation of the total intelligence quotient (IQ) through the standardized administration of scales, including verbal subtests to determine the verbal quotient (VQ), and performance subtests to determine performance quotient (PQ) [31].

### Statistical analyses

Results are shown as median with interquartiles. The Kolmogorov–Smirnov test was utilized to assess the normality of parameter distribution. Mean values were compared by the unpaired Student *t*-test in parameters with normal distribution, and linear correlations evaluated with Pearson’s correlation coefficient. Significance was calculated with the Mann–Whitney test and Spearman’s correlation coefficient in parameters with skewed distribution. Multiple regression analyses were applied to identify the relative strength of each biochemical and clinical variable in predicting irisin levels. The Statistical Package for the Social Sciences (SPSS) for Windows, version 22.0 (SPSS Inc., Chicago, IL, USA) was utilized for statistical analysis. The limit of statistical significance was set at 0.05.

## Results

### Linking irisin levels to the genetic background and metabolic profile and bone health in pediatric and adult PWS

**Fig. 1:**
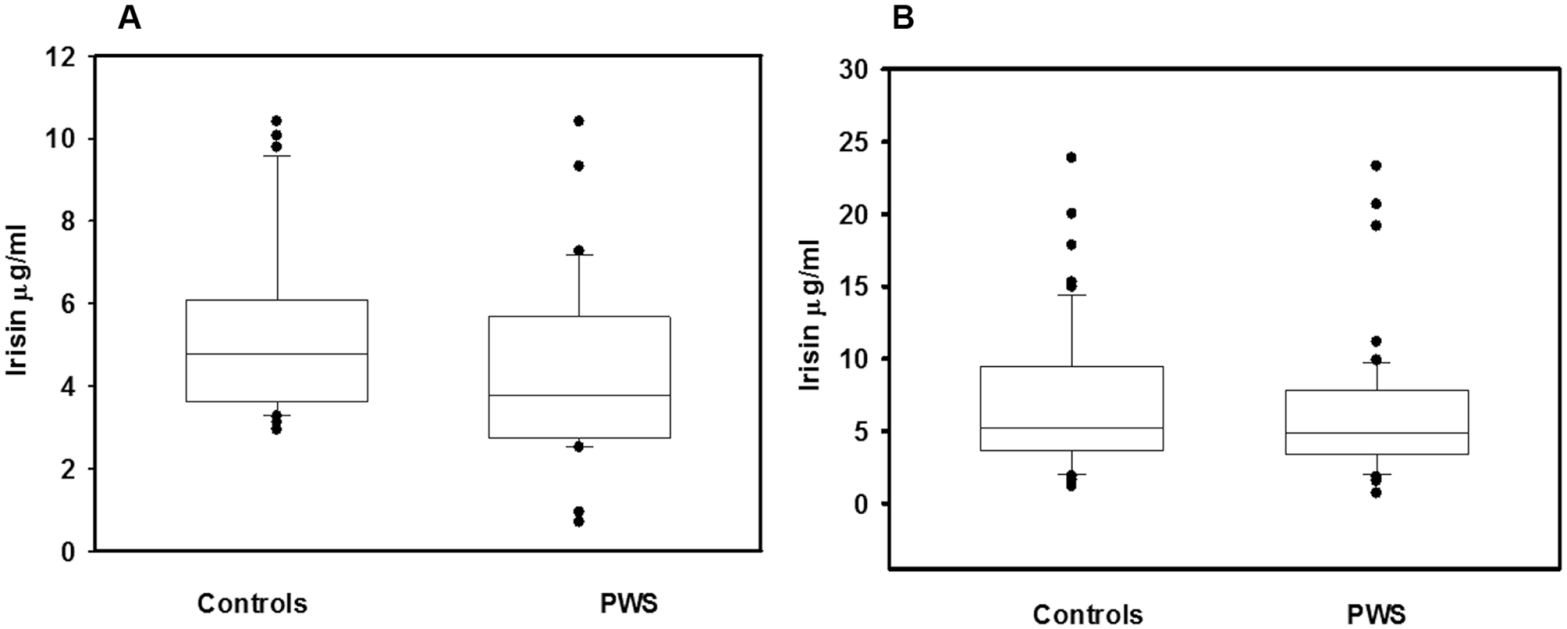
Irisin levels in PWS subjects. Mean serum irisin levels did not change significantly between PWS children and controls (4.37 ± 2.30 μg/ml vs 5.31 ± 2.13 μg/ml) (**a**) as well as between adult PWS and controls (6.65 ± 4.49 μg/ml vs 7.24 ± 5.20 μg/ml) (**b**)

**Fig. 2:**
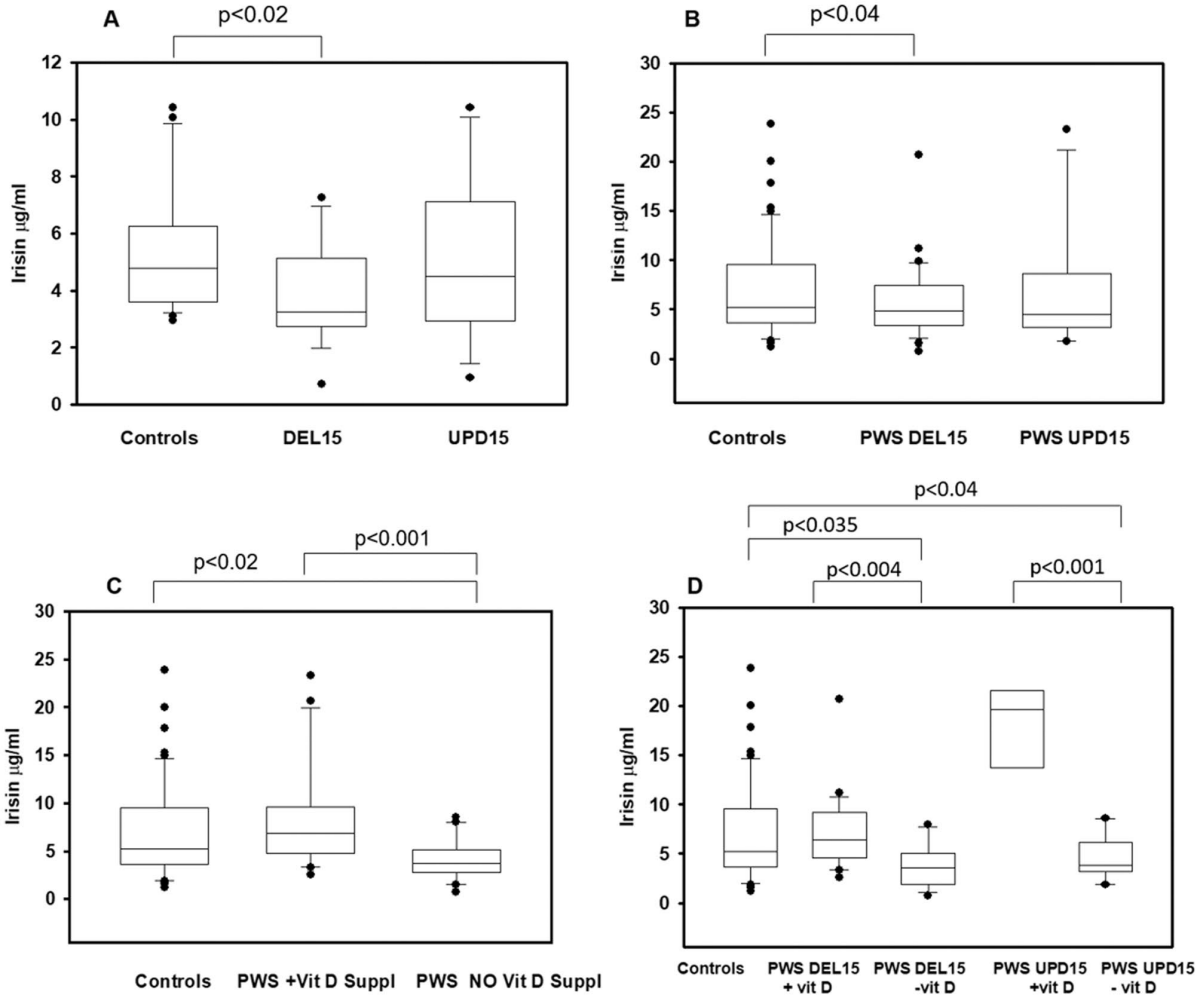
Linking irisin levels to the genetic background and Vitamin D supplementation. Pediatric (**a**) and adult (**b**) PWS with DEL15 showed significantly reduced irisin levels compared with the controls (*p* < 0.02 and *p* < 0.04, respectively), but not UPD15. Adult PWS patients (**c**) without vitamin D supplementation showed a significant reduction of the myokine levels compared with the controls and the patients performing the supplementation (*p* < 0.001 and *p* < 0.02). The lowest levels of irisin are associated to the lacking of vitamin D supplementation for both DEL15 and UPD15 groups (*p* < 0.004 and *p* < 0.001) (**d**)

Clinical and biochemical characteristics of PWS patients are reported in Table 1. Mean serum irisin levels did not change significantly between PWS children and controls (4.37 ± 2.30 μg/ml vs 5.31 ± 2.13 μg/ml, respectively) as well as between adult PWS and controls (6.65 ± 4.49 μg/ml vs 7.24 ± 5.20 μg/ml, respectively) (Fig. 1). A more in-depth analysis showed that the type of genetic alteration in PWS patients affected irisin levels. In fact, both pediatric and adult PWS with DEL15 showed significantly reduced irisin levels compared with the controls (*p* < 0.02 and *p* < 0.04, respectively) (Fig. 2a, b). Otherwise, pediatric and adult PWS with UPD15 did not display significant differences compared with the controls (Fig. 2a, b). These findings prompted us to evaluate if there were significant differences among clinical, biochemical and bone parameters according to the genetic background.

**Table 1:** Clinical and biochemical characteristics of study population

	Pediatric PWS, *N* = 26	Pediatric controls, *N* = 26	Adult PWS, *N* = 52	Adult controls, *N* = 54
Auxological parameters				
Gender (male/female)	11/15	9/17	22/30	28/26
Age (years)	10.86 (6)	9.40 (3.29)	36.00 (20.00)	36.9 (18.5)
Tanner (I/II/III/IV/V)	23/1/2/0/0	22/2/2/0/0	0/0/25/25/2	0/0/0/0/54
Height SDS	− 0.70 (1.68)	0.40 (1.21)	–	–
Weight SDS	1.00 (1.95)	0.53 (0.95)	–	–
BMI	25.27 (8.4)	23.4 (0.92)	35.3 (12.1)^§^	24.5 (2.1)
BMI-SDS	2.03 (1.76)	0.30 (0.82)	–	–
FM (g)	23,756 (8388)	–	39,150 (16,311)	–
FM (%)	41.05 (12.7)	–	50.50 (10.50)	–
FFM (g)	28,913 (9358)	–	41,582 (10,181)	–
FFM (%)	55.93 (11.4)	–	49.50 (9.00)	–
Total IQ	71.00 (20.00)	–	57.00 (11.00)	–
Verbal IQ	-	–	58.00 (11.00)	–
Performance IQ	–	–	66.00 (17.00)	–
Laboratory parameters				
Total cholesterol (mg/dl)	159.9 (45.0)	144.3 (18.6)	180.0 (52.0)	
LDL–C (mg/dl)	94.00 (27.0)	78.1 (20.2)	119.0 (42.0)	
HDL-C (mg/dl)	54.00 (16.00)	57.8 (8.6)	48.0 (20.0)	
Triglycerides (mg/dl)	64.00 (50.00)	59.2 (15.4)	95.0 (51.0)	
Glucose (mg/dl)	78.00 ( 9.00)	87.22 (11.35)	90.0 (19.0)	
Insulin (mU/l)	8.49 (6.71)	9.2 (3.8)	9.6 (5.51)	
HOMA-index	1.61 (1.32)	1.62 (0.8)	2.15 (1.70)	
Osteocalcin (ng/ml)	89.00 (14.35)*	38.3 (19.2)	19.00 (14.00)	
PTH (pg/ml)	43.00 (34.00)	19.25 ( 5.1)	49.4 (34.3)	
Ca (mg/dl)	9.80 (0.70)	9.71 (0.40)	9.50 (0.60)	
P (mg/dl)	4.70 (0.80)	4.54 (1.40)	3.90 (0.70)	
25 (OH) vitamin D	23.17 (6.70)		28.8 (9.2)	
Bone parameters				
LS-BMD *Z* score	0.58 (1.51)		–	–
LS-BMD *T* score			− 1.10 (0.20)	–
TBLH BMC (g)	1415 (329)		2096 (13.09)	
TBLH BMD (g/cm^2^)	0.91 (0.16)		1.18 (0.08)	
LS BMC (g)	30.76 (9.68)		56.27 (1.17)	
LS BMD (g/cm^2^)	0.71 (0.28)		1.05 (0.06)	
LSBMAD (g/cm^3^)	0.14 (0.04)		0.18 (0.01)	
TBLH BMD-Ht (g/cm^3^)	0.63 (0.08)		0.75 (0.07)	

*PWS* Prader–Willi syndrome, *SDS* standard deviation score, *FM* fat mass, *FFM* fat free mass, intelligence quotient (IQ) , *BMI* body mass index, *LDL-C* low-density lipoprotein cholesterol, *HDL-C* high-density lipoprotein cholesterol, *HOMA* *index* homeostasis model assessment index, *PTH* parathyroid hormone, *Ca* calcium, *P* phosphorus, *LS* lumbar spine, *TBLH* total body less head, *BMD* bone mineral density, *BMAD* bone mineral apparent density, *BMD-Ht* height adjusted

*p < 0.05 pediatric PWS with respect to pediatric controls

^§^*p* < 0.05 adult PWS with respect to adult controls

We found that in the pediatric population only total cholesterol and LDL-C statistically differs between DEL15 and UPD15 (Table 2). About bone densitometric parameters, although there was a trend toward the reduction in UPD15 patients compared with DEL15 patients, this did not reach the statistical significance (Table 2). Interestingly, higher level of 25(OH) vitamin D were measured in UPD15 patients compared with DEL15 patients, but the difference did not achieve the statistical significance (Table 2).

**Table 2:** Clinical and biochemical characteristics of PWS patients according to genetic background

	Pediatric DEL PWS, *N* = 16	Pediatric UPD PWS, *N* = 10	Adult DEL PWS, *N* = 38	Adult UPD PWS, *N* = 14
Auxological parameters				
Gender (male/female)	4/12	6/4	20/18	5/9
Age (years)	9.82 (5)	10.71 (8.65)	28.64 (22.00)	21.59 (5.43)
Height SDS	− 0.66 (2.00)	− 0.38 (2.60)	–	–
Weight SDS	1.23 (2)	1.22 (2.18)	–	–
BMI			34.30 (15.00)	32.34 (10.94)
BMI-SDS	1.92 (2)	2.13 (3.82)	–	–
Total IQ	71.00 (10.1)	69.00 (3.6)	58.17 (19.2)	54.00 (10.00)
Verbal IQ	–	–	61.00 (18.75)	57.00 (7.50)
Performance IQ	–	–	63.50 (19.25)	68.00 (18.50)
Laboratory parameters				
Total cholesterol (mg/dl)	148.0 (44)*	174.5 (42.25)	135.00 (28.00)	158.0 (16.00)
LDL-C (mg/dl)	92 (34)*	96.5 (32.25)	70.00 (48.00)	96.00 (12.00)
HDL-C (mg/dl)	54 (22)	53.00 (24.50)	47.00 (14.00)	47.00 (17.00)
Triglycerides (mg/dl)	58 (41)	83.00 (69.7)	70.00 (36.00)	67.00 (40.00)
Glucose (mg/dl)	77.50 (7.00)	81.5 (16.5)	85.00 (18.00)	78.00 (39.00)
Insulin (mU/l)	9.17 (7.00)	10.08 9.71)	8.50 (5.55)	10.51 (3.39)
HOMA-index	1.71 (1.00)	2.06 (2.09)	1.52 (0.67)	1.60 (1.82)
Osteocalcin (ng/ml)	71.74 (26.42)	91.00 (34.00)	19.00 (16.00)	12.0 (4.8)
PTH (pg/ml)	62 (36)	35.00 (19.88)	49.40 (23.6)	26.00 (17.75)
Ca (mg/dl)	10 (1)	9.55 0.37)	9.30 (0.30)	9.60 (0.36)
P (mg/dl)	4.80 (1)	4.85 (1.15)	3.60 (0.60)	4.20 (0.41)
25 (OH) vitamin D (ng/ml)	22.60 (5.00)	26.1 (4.65)	31.60 (9.20)	28.7 (10.5)
Bone parameters				
LBMD *Z* score	0.69 (2.00)	0.40 (1.00)	–	–
LS-BMD *T* score	–	–	− 1.00 (1.60)	− 0.30 (1.02)
TBLH BMC (g)	1134 (299)	1201 (225)	2111 (274)	2083 (93)
TBLH BMD (g/cm^2^)	0.83 (0.08)	0.87 (0.11)	1.10 (0.05)	1.10 (0.05)
LS BMC (g)	31.85 (10.01)	6.12 (5.51)	59.49 (17.00)	56.27 (9.43)
LS BMD (g/cm^2^)	0.71 (0.12)	0.66 (0.10)	0.99 (0.07)	0.99 (0.07)
LS BMAD (g/cm^3^)	0.16 (0.02)	0.13 (0.02)	0.17 (0.009)	0.67 (0.007)
TBLHBMD-Ht	0.63 (0.02)	0.62 (0.13)	0.67 (0.06)	0.70 (0.06)

*PWS* Prader–Willi syndrome, *SDS* standard deviation score, *IQ* intelligence quotient, *BMI* body mass index, *LDL-C* low-density lipoprotein cholesterol, *HDL-C* high-density lipoprotein cholesterol, *HOMA index* homeostasis model assessment index, *PTH* parathyroid hormone, *Ca* calcium, *P* phosphorus, *LS* lumbar spine, *TBLH* total body less head, *BMD* bone minal density, *BMAD* bone mineral apparent density, *BMD-Ht* height adjusted

*p < 0.02 pediatric DEL PWS with respect to pediatric UPD PWS

Furthermore, we found that adult PWS patients performing vitamin D supplementation had irisin levels similar with controls, whereas adult PWS patients without vitamin D supplementation showed a significant reduction of the myokine levels compared with the controls and the patients performing the supplementation (*p* < 0.001 and *p* < 0.02, respectively), Fig. 2c. Consistently, adult PWS patients performing vitamin D supplementation showed significantly higher levels of 25(OH) vitamin D compared with PWS patients not performing the supplementation (33.39 ± 9.87 vs 27.03 ± 6.04, *p* < 0.05). This issue was not investigated in pediatric PWS as only four performed vitamin D supplementation.

Finally, if we consider together the vitamin D supplementation and the genetics of adult PWS patients, the lowest levels of irisin are associated to the lacking of vitamin D supplementation for both DEL15 and UPD15 groups (*p* < 0.004 and *p* < 0.001, respectively) (Fig. 2d). Consistently, patients performing vitamin D supplementation showed high levels of circulating 25(OH) vitamin D compared with patients not performing the supplementation (DEL15 30.52 ± 9.17 vs 24.34 ± 5.60, *p* < 0.04; UPD15 36.98 ± 16.58 vs 30.62 ± 4.77, respectively).

### Correlations and multiple regression analysis among irisin levels and anthropometric and metabolic parameters as well as cognitive performance and bone mineral density

Table 3 shows the correlations between irisin levels and anthropometric, metabolic parameters, cognitive performance and instrumental parameters of bone health in our study population. In pediatric PWS subjects the irisin levels positively correlated with BMI-SDS, weight-SDS, height-SDS, FM, FM%, glucose, insulin, HOMA-IR, vitamin D dosage supplementation, and 25(OH)-vitamin D levels; otherwise, the irisin levels negatively correlated with HDL, FFM%, calcium, and PTH. In PWS adults the irisin levels correlated positively with cholesterol, HDL, FM, FM%, years of GH therapy, glucose, age of sex steroid replacement therapy, age at start of GH therapy, LS-T-score, TBLH BMD-Ht, IQ, verbal IQ and performance IQ. Otherwise, a negative correlation was found between irisin levels and 25(OH)-vitamin D levels, FFM%, calcium, and PTH.

**Table 3:** Significant correlations of irisin levels with anthropometric, metabolic, cognitive and instrumental parameters

	Irisin (pediatric PWS)
BMI-SDS	*r* = 0.562, *p* < 0.0001
Weight-SDS	*r* = 0.682, * p* < 0.0001
Height-SDS	*r* = 0.390, * p* < 0.0001
FM (g)	*r* = 0.500, * p* < 0.0001
FM (%)	*r* = 0.384, * p* < 0.001
FFM (%)	*r* = − 0.384, * p* < 0.001
LS-BMD *Z* score	*r* = 0.137, * p* < 0.049
Glucose	*r* = 0.282, * p* < 0.0001
Insulin	*r* = 0.388, * p* < 0.0001
HOMA-IR	*r* = 0.257, * p* < 0.0001
25(OH) vitamin D	*r* = 0.329, * p* < 0.0001
HDL	*r* = − 0.313, * p* < 0.0001
Calcium	*r* = − 0.177, * p* < 0.0001
PTH	*r* = − 0.405, * p* < 0.0001

	Irisin (adult PWS)
Cholesterol	*r* = 0.266, * p* < 0.0001
LDL	*r* = 0.220, * p* < 0.0001
HDL	*r* = 0.416, * p* < 0.005
FM (g)	*r* = 0.099, * p* < 0.001*
FM (%)	*r* = 0.135, * p* < 0.0001*
FFM (%)	*r* = − 0.083, * p* < 0.004*
25 (OH) vitamin D	*r* = − 0.103, * p* < 0.0001
Years of GH therapy	*r* = 0.568, * p* < 0.02
Vitamin D supplementation	*r* = − 0.383, * p* < 0.0001
LS BMD *T* score	*r* = 0.262, * p* < 0.0001
TBLH BMD-Ht	*r* = 0.064, * p* < 0.027
Glucose	*r* = 0.205, * p* < 0.0001
Age of sex steroid replacement therapy	*r* = 0.361, * p* < 0.0001
Age of GH therapy	*r* = 0.328, * p* < 0.0001
Calcium	*r* = − 0.084, * p* < 0.001
PTH	*r* = − 0.203, * p* < 0.0001
IQ	*r* = 0.372, * p* < 0.04
Verbal IQ	*r* = 0.196, * p* < 0.0001
Performance IQ	*r* = 0.289, * p* < 0.0001

Additionally, multiple linear regression analyses were performed to explore the factors affecting irisin levels in PWS patients. Multiple linear regression analysis for irisin as dependent variable demonstrated that weight-SDS, genetics, 25(OH)-vitamin D levels and LS BMD *Z* score were the most important predictors in pediatric PWS subjects (Table 4). With adjustment for age, in adult PWS the best predictors for irisin levels were the genetic background, 25(OH)-vitamin D levels, GH therapy, the age at start and the duration of GH treatment, the age at start of sex steroid replacement therapy, IQ and TBLH BMD-Ht.

**Table 4:** Multiple regression analysis for pediatric and adult PWS

	Dependent variable	Independent variable	*β*	*p*	*r*
Model 1 (children)					
				0.0001	0.944
	Irisin	Weight-SDS	0.298	0.0001	
		Genetics	−0.802	0.0001	
		25 (OH) vitamin D	0.050	0.199	
		LS *Z* score	0.147	0.009	
Model 2 (adults)					
				0.0001	0.665
	Irisin	Genetics	−0.382	0.0001	
		25 (OH) vitamin D	0.338	0.0001	
		GH therapy	0.370	0.0001	
		Age for GH therapy	0.694	0.0001	
		Age for sex steroid replacement treatment	−0.128	0.0001	
		Total BMD	0.378	0.0001	
Model 3 (adults)					
				0.0001	0.750
	Irisin	Genetics	−0.365	0.0001	
		25 (OH) vitamin D	0.346	0.0001	
		GH Therapy	−0.139	0.0001	
		Age for GH Therapy	0.317	0.0001	
		Age for sex steroid replacement treatment	−0.324	0.0001	
		Total BMD	0.412	0.0001	
		Total IQ	0.910	0.0001	

## Discussion

The present study displayed that PWS patients has comparable levels of irisin with respect to the controls; interestingly, a deepened analysis showed that both pediatric and adult PWS with DEL15 have significantly reduced levels of irisin compared with the controls, suggesting that the genetic background could be associated with a different metabolic profile in PWS [32]. Additionally, we also showed that patients who did not receive vitamin D supplementation had low serum levels of irisin, despite having the UPD as genetic alterations. To our knowledge, this is the first study, which evaluated irisin levels both in PWS children and adults. Previous studies assessed irisin levels only in adult PWS patients. In detail, Hirsch et al. found higher levels of irisin in the saliva of PWS patients than controls, probably due to the different composition, and not significant differences in plasma levels between the two groups [17]. The same authors found that in PWS patients and controls plasma irisin levels positively correlated with total cholesterol and LDL, whereas in the saliva the myokine levels was inversely related with HDL, and directly with LDL and triglycerides [17]. Recently, the same research group again demonstrated that the serum levels of the myokine did not change in adult PWS compared with the controls, even if they performed a resistance exercise [18]. These results can be explained by the hypotonic muscle mass of these subjects.

Conversely, we found that irisin serum levels directly correlated with HDL in PWS children, whereas a positive correlation between irisin levels and total cholesterol, LDL, and HDL, but not with triglycerides was found in PWS adults. Recently, Mai et al. demonstrated that obese PWS adults showed comparable levels of irisin with respect to controls, but lower irisin amounts than obese subjects [19]. Interestingly, the authors also reported that in PWS patients irisin levels correlated with triglycerides [19]. This finding may be related to the peculiar body composition of PWS, characterized by lower visceral adipose tissue and decreased muscle mass [33], as well as to the impairment of adipose tissue observed in these subjects [34]. Consistently, we also found that irisin level correlated with FM and FFM. In agreement with our data, Mai et al. reported that a positive correlation was evident between irisin and FM% after adjustment for the PWS status [19]. On the other hand, our data showed that in the pediatric population the levels of the myokine correlated with BMI-SDS and weight-SDS, as well as parameters of glycemic and lipid metabolism.

Our paper is the first to demonstrate a direct correlation between the levels of this myokine and LS-BMD *Z* score and LS-BMD *T* score in pediatric and adult PWS, respectively. The anabolic role of irisin on bone has been demonstrated in healthy and osteoporotic mice [11, 12]. In humans, irisin correlates negatively with the serum levels of sclerostin, an inhibitor of Wnt β-catenin pathway [35]. Moreover, a direct correlation of the myokine with bone strength and BMD has been demonstrated in athletes [36] as well as in soccer players [37]. We also reported a positive association between bone status and serum irisin levels in healthy and diabetic children [38, 39]. In our population of pediatric and adult PWS subjects the irisin levels were negatively related to PTH. Consistently, in vitro experiments demonstrated a negative relationship between PTH and irisin, and these findings were further supported by the reduced concentration of the myokine in post-menopausal women with primary hyperparathyroidism with respect to the controls [40]. Although it has been reported that irisin levels were associated with osteoporotic fractures [41], in our study population previous post-traumatic fractures have been described only in five adult PWS patients, and thus it was not possible to evaluate the statistic relevance.

Interestingly, our study revealed that in pediatric PWS the vitamin D levels affected irisin serum concentration. This finding is a novelty in pediatric PWS population as previously we did not find significant correlation between irisin and 25(OH) vitamin D levels in healthy children [38]. The most of our pediatric PWS subjects showed normal vitamin D levels, thus they did not perform vitamin D supplementation, in contrast with our adult PWS population. Indeed, we demonstrated that irisin serum levels were reduced in adult patients without vitamin D supplementation, suggesting that vitamin D supplementation has an important role in regulating irisin levels in adult PWS patients. Consistently, it has been recently reported that vitamin D supplementation improves irisin levels in obese type 2 diabetic patients [42].

In adult PWS patients we demonstrated a direct link with the age of sex steroid replacement therapy and the age of GH therapy, suggesting the key role of the beginning of the therapy to normalize the levels of the myokine in these subjects. Literature data reported the strict connection between irisin levels and GH as well as the favorable effect of GH replacement therapy on the myokine levels in children with GH deficiency [43].

Interestingly, irisin has been linked to cognitive impairment and neurodegenerative diseases [44], and in adults at risk of dementia its levels correlated with global cognition [45], thus irisin also could represent a serum biomarker of cognitive impairment. Although the results are referred only to 29 adult PWS, we showed that the levels of the myokine positively correlated with total IQ, verbal IQ and performance IQ. However, we did not find the same results for pediatric PWS, thus it is possible that it was evident only in adults, because the IQ impairment was more serious, but also maybe because we had total IQ evaluation only for a restricted number of pediatric subjects. Consequently, this issue will require future investigations with larger study cohorts.

In conclusion, we did not find different irisin levels in PWS patients compared to matched controls, but we demonstrate possible role of genetic background in PWS on irisin level. Vitamin D supplementation may be key factor in regulating serum irisin levels.
